# Intermittent theta-burst stimulation in aphasia caused by right side cerebral lesions after stroke: A case report with 2-year follow-up

**DOI:** 10.1016/j.heliyon.2024.e35206

**Published:** 2024-07-25

**Authors:** Haozheng Li, Shunjuan Fan, Yi Wu, Dongxiang Fang, Ruiping Hu, Rongrong Lu

**Affiliations:** a Department of Rehabilitation Medicine, Huashan Hospital, Fudan University, 200040, Shanghai, China; b National Center for Neurological Disorders, 20040, Shanghai, China

**Keywords:** Stroke, Aphasia, Intermittent theta-burst stimulation, Functional near-infrared spectroscopy, Neural plasticity

## Abstract

**Background and objectives:**

This case report investigates the application of intermittent Theta-Burst Stimulation (iTBS) in aphasia rehabilitation following a right hemisphere stroke.

**Case presentation:**

A 52-year-old Chinese male with Broca's aphasia post-stroke was treated with iTBS. His progress was evaluated using Functional Near-Infrared Spectroscopy (fNIRS) and behavioral assessments. Significant language function improvement was noted, with fNIRS showing increased activation in right hemisphere language-related cortical areas and altered functional connectivity patterns.

**Conclusion:**

The findings indicate that iTBS is effective in facilitating language recovery in right hemisphere stroke-induced aphasia, highlighting the importance of personalized neurorehabilitation strategies. Despite focusing on a single case, the study contributes to understanding neural plasticity mechanisms in right hemisphere stroke-induced aphasia.

## 1 Introduction

Aphasia is characterized by the loss or impairment of language and speech functions, affecting approximately 24%–50 % of stroke patients [1]. It can manifest as difficulties in speaking, comprehension, repetition, and naming, significantly impairing the patients' communication abilities and making it challenging for them to reintegrate into society [2]. The majority of aphasia cases are attributed to lesions on the left hemisphere. About 95 % of right-handed individuals and 76 % of left-handed individuals have their dominant language hemisphere on the left side [2]. Aphasia due to right hemisphere lesions is rare, with only a few cases reported. Crossed aphasia is thought to occur in right-handed patients with right hemisphere damage, yet their primary language centers remain in the left hemisphere, accounting for approximately 3 % of all aphasia cases [3].

Large-sample studies have indicated that despite active language rehabilitation training, approximately 20 % of post-stroke aphasia patients are unable to recover even the most basic daily communication abilities [4]. Transcranial Magnetic Stimulation (TMS) is an emerging non-invasive brain stimulation technique. Excitatory TMS can function by reducing local GABA-ergic inhibition and by promoting long-term potentiation effects [5]. Intermittent Theta Burst Stimulation (iTBS), a popular novel excitatory TMS intervention modality with burst frequencies typically around 50Hz, has been shown to rapidly induce neuronal excitation effects in stimulated brain regions [6,7]. It has been demonstrated to improve language performance in post-stroke aphasia patients through prolonged intervention [8,9].

In this case report, we report a patient with aphasia resulting from a right hemisphere stroke. We hypothesized that long-term intervention could significantly enhance and sustain his language abilities. Long-term follow-up and functional imaging techniques were employed to capture the neural plasticity process in his language-related cortical areas. To our knowledge, no studies to date have applied iTBS intervention to rare cases of aphasia caused by right hemisphere damage, nor observed the neural plasticity process in the language-related cortices of such patients over an extended period.

## 2 Case presentation

### 2.1 Diagnostic assessments

**Fig. 1:**
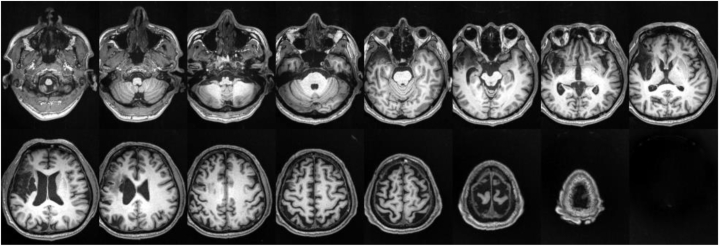
MRI of the patient, the brain lesion was predominantly located in his right hemisphere.

An ambidextrous 52-year-old male Chinese native speaker was referred to our rehabilitation clinic one month after suffering a stroke resulting in acute cerebral infarction in the right middle cerebral artery region (as shown in Fig. 1). He was diagnosed as Broca's aphasia and left hemiplegia, the Edinburgh Inventory is a commonly used tool to assess individuals' hand preference for activities such as writing, throwing balls, and using tools [10]. His score on the Edinburgh Inventory was 20, indicating mixed-handedness (scores range from −40 to 40: <-40 indicates left-handedness, −40 to 40 indicates mixed-handedness, and >40 indicates right-handedness). The Western Aphasia Battery (WAB) is a comprehensive test used to evaluate language abilities in individuals with aphasia, including tasks assessing fluency, comprehension, naming objects and pictures, and repeating phrases [11]. His Aphasia Quotient on the WAB (Simplified Chinese version) was 47.2/100, indicating moderate aphasia (Aphasia Quotient ranges from 0 to 100: less than 31.3 indicates severe aphasia, 31.3 to 62.5 indicates moderate aphasia, 62.6 to 93.7 indicates mild aphasia, and above 93.8 suggests negligible aphasia). The Non-language-based Cognitive Assessment (NLCA) assesses cognitive abilities independent of language, using visual or abstract stimuli to evaluate reasoning, spatial ability, memory, and problem-solving skills such as pattern recognition, shape matching, completing puzzles, and understanding spatial relationships [12]. His NLCA score was 77/80, indicating near-normal non-verbal cognitive functioning with only minor impairment (scores below 70 indicate significant cognitive impairment, while scores between 70 and 80 suggest mild or suspected cognitive impairment). Detailed information is provided in Table 1.

**Table 1:** Test scores of WAB and NLCA.

	Baseline 0 Month	Treatment 1 Month	After treatment 3 Month	Follow-up 2 Year
Aphasia quotient	47.2/100	75.5/100	92.8/100	97.4/100
Spontaneous speech	10/20	13/20	17/20	19/20
Auditory comprehension	6.8/10	9.65/10	10/10	10/10
Repetition	4.4/10	9.2/10	10/10	10/10
Naming	2.4/10	5.9/10	9.4/10	9.7/10
NLCA(Total)	77/80	80/80	80/80	80/80
Memory	19/20	20/20	20/20	20/20
Attention	30/30	30/30	30/30	30/30
Reasoning	7/8	8/8	8/8	8/8
Executive functions	9/9	9/9	9/9	9/9
Visuospatial functions	12/13	13/13	13/13	13/13

We employed language function scales and fNIRS scanning to monitor his functional recovery and neural plasticity. The experiment was conducted in accordance with the Helsinki Declaration, and the patient was provided with written a informed consent form.

### 2.2 Diagnostic assessments

**Fig. 2:**
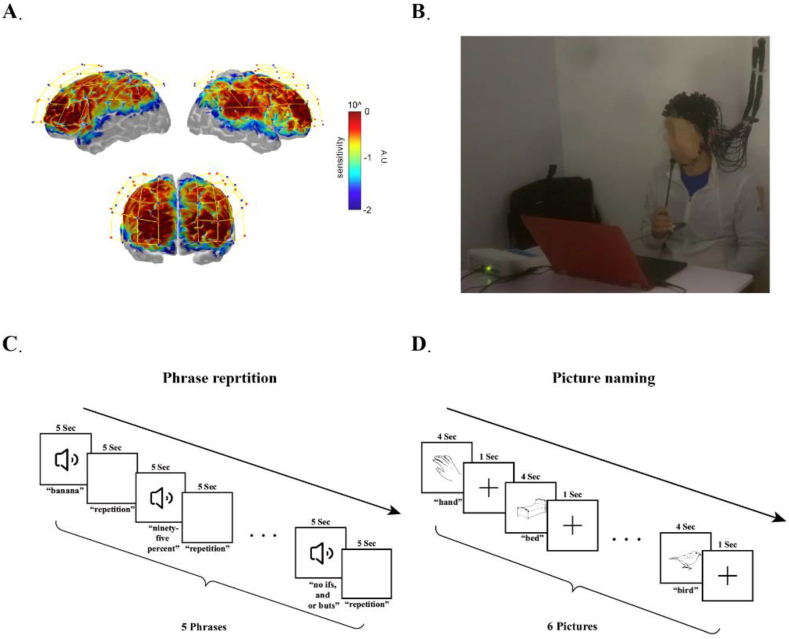
(A) Sensitivity profile, sources are displayed with red dots while detectors are displayed with blue dots and channels with yellow lines. Results of the Monte-Carlo simulation based on 1 × 10-8 photons. (B) Scenarios for fNIRS scanning (C) Phrase repetition task. (D) Picture naming task.

A continuous wave fNIRS device (NirSmart-6000A) was utilized to collect fNIRS data (refer to Fig. 2A and B). We gathered 8 min of resting-state fNIRS data from the patient, during which they were instructed to sit quietly with their eyes closed, remaining relaxed but awake. The language-related tasks we employed our previously established fNIRS research paradigm, used with Chinese-speaking aphasia patients [13]. We used a block design, consisting of experimental blocks (30 seconds) and control blocks (20 seconds) during the picture naming task, wherein patients were asked to name images displayed on a screen. Similarly, we designed the phrase repetition task with experimental blocks lasting 50 seconds, and control blocks lasting 30 seconds. During the experimental blocks, the patient was instructed to loudly repeat words played through headphones (see Fig. 2C and D). The patient underwent a total of four fNIRS scanning sessions, corresponding to baseline (0 Month), during treatment (1 Month), post-treatment (3 Months), and follow-up (2 Years).

### 2.3 Therapeutic interventions

We implemented an iTBS protocol targeting the right inferior frontal gyrus area of the patient's brain. Concurrently, the patient was receiving speech therapy, each lasting 30 minutes, conducted five times a week. This combined therapeutic intervention spanned a total duration of three months.

### 2.4 Outcome

For the fNIRS data, we analysed it using Homer2 software, focusing on the functional connectivity within and between the language-related cortical areas (Broca's and Wernicke's areas) in both hemispheres. Additionally, we utilized NIRS_SPM software to estimate the magnitude of activation values in language-related task data. Hemisphere asymmetry of activation was determined using a Laterality Index (LI), defined as [(ΔHbO in Affected Hemisphere – ΔHbO in Unaffected Hemisphere)/(ΔHbO in Affected Hemisphere + ΔHbO in Unaffected Hemisphere)]. Laterality indexes ranging from 0 to 1 and -1 to 0 indicated activation in the lesional and non-lesional hemispheres, respectively [14].

**Fig. 3:**
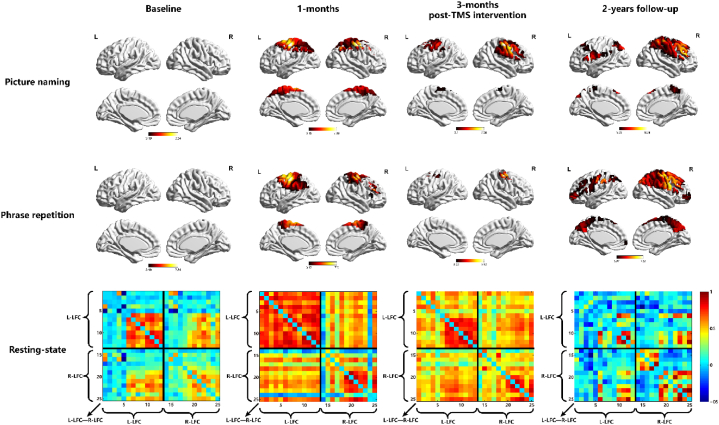
Bright colours in the task-state images denote regions of increased brain activation (p < 0.05). In the resting-state images, functional connectivity was categorized into three areas: the left language-related cortex, the right language-related cortex, and the functional connectivity between the left and right cortices. These areas are outlined by a black box. Warm colours represent a higher strength of functional connectivity, while cool colours signify a lower strength of functional connectivity.

At baseline, the patient was in the acute phase, exhibiting low scores on language scales. Both naming and repetition tasks revealed low activation in bilateral language-related cortical areas and bilateral dominance. The functional connectivity of these language-related cortices was also at a lower level. After one month of iTBS intervention, during the subacute phase, both naming and repetition tasks showed increased activation in bilateral language-related cortices, with a marked left-lateralized dominance. The functional connectivity in bilateral language-related cortices was enhanced, particularly in the left hemisphere, and the patient's language scale scores were significantly improved. Following three months of iTBS intervention, there was a reduction in task-related activation in bilateral language-related cortices, shifting towards right hemisphere dominance. While the functional connectivity in bilateral language-related cortices remained high, there was a decrease in the left hemisphere and an increase in the right. The patient's scale scores were further improved, nearing those typical of non-aphasic individuals (Table 1, Table 2, and Fig. 3).

**Table 2:** The result of fNIRS Scanning.

	Baseline 0 Month	Treatment 1 Month	After treatment 3 Month	Follow-up 2 Year
LI(Naming)	0.056	−0.193	0.217	0.478
LI(Repetition)	0.062	−0.223	0.315	0.367
FC(L-LFC)	0.259	0.735	0.215	0.095
FC(R-LFC)	0.257	0.394	0.488	0.211
FC(LLFC-RLFC)	0.212	0.423	0.466	0.098

Note: LI, laterality index; FC, functional connectivity; LFC, language-related cortices.

### 2.5 Follow-up

At the two-year follow-up post-intervention, now in the chronic phase, the patient displayed high activation in bilateral language-related cortices during language tasks with a strong rightward bias. The functional connectivity between and within bilateral language-related cortices had been decreased, with a higher connectivity in the right hemisphere. At this point, the patient's language function could no longer be classified as aphasic (see Table 1, Table 2, and Fig. 3).

## 3 Discussion

In this case report, we identified the stimulation target for language improvement in a patient with aphasia resulting from a right hemisphere stroke. By applying prolonged iTBS intervention, the patient's language function showed a significant improvement, which was maintained during long-term follow-up. We employed fNIRS neuroimaging technology to observe the longitudinal reorganization in the language-related cortical areas. Initially, in the acute phase, we exhibited low activation in the language-related cortices from the patient with bilateral lateralization and reduced functional connectivity. In the subacute phase, there was a marked increase in activation in these cortices, predominantly left-lateralized, which gradually shifted to the right side. High functional connectivity was observed, later transitioning from left to right. During the chronic phase, extensive bilateral activation in the language-related cortices was noted, primarily right-lateralized, with reduced functional connectivity and a right hemisphere dominance.

Unexpectedly, the patient in our reported case exhibited rapid recovery following prolonged iTBS intervention. Three months post-TMS intervention, the patient's WAB score approached that of non-aphasic individuals and remained so over the long term. This may happen due to the extended iTBS treatment amplifying the beneficial effects of single interventions in both magnitude and duration, fostering neuroplasticity in the damaged cortex, aiding neural regeneration in the language-related areas of the impaired hemisphere, and translating these changes into sustained improvements in language performance. One study reported that, following prolonged iTBS intervention in post-stroke aphasia patients, Diffusion Tensor Imaging scans showed increased fractional anisotropy scores near the intervention target (near the inferior and superior frontal gyri), possibly reflecting iTBS-mediated improvement in cortical function through enhanced synaptic connectivity [15]. Several Randomized Controlled Trial studies have also demonstrated that long-term iTBS intervention on the impaired hemisphere can significantly improve language functions in post-stroke aphasia patients [8,16]. Current functional neuroimaging research suggest that activation of the impaired hemisphere plays a crucial role in the recovery of language functions in post-stroke aphasia patients [17,18]. In a pilot study involving eight post-stroke aphasia patients, prolonged iTBS treatment targeting the residual area of the inferior frontal gyrus in the impaired hemisphere induced a shift in the Laterality Index, from the impaired to the unaffected hemispheres's inferior frontal gyrus. This increased activation in the impaired hemisphere's inferior frontal gyrus and decreased activation in the unaffected hemispheres's counterpart. Changes in activation in the unaffected hemispheres's inferior frontal gyrus were correlated with improvements in the patients' language performance [19]. These findings align with our study results, where, despite the patient's lesion being in the right hemisphere, fNIRS scanning indicated increased activation and lateralization in the impaired hemisphere over the course of treatment, concurrent with the recovery of language function.

Another key finding was that, in the acute phase, the patient exhibited low activation in language-related cortices with reduced bilateral lateralization and functional connectivity. In the subacute phase, there was a marked increase in activation of these cortices, primarily left-lateralized, gradually transitioning to the right side. We observed high functional connectivity, subsequently shifting from the left to the right. In the chronic phase, language-related cortices showed extensive bilateral activation, predominantly on the right, with reduced functional connectivity and right hemisphere dominance. Saur and colleagues [20,21], through two large-sample long-term neuroimaging studies of post-stroke aphasia patients, revealed a pattern of neural reorganization over time: in the acute phase, reduced bilateral hemisphere activation showed only slight activation; in the subacute phase, increased bilateral activation had peak activation in the unaffected hemispheres; however, in the chronic phase, activation shifted from undamaged to damaged hemisphere. Our case report's fNIRS scanning results support his hypothesis. Although the patient's lesion was in the right hemisphere, as opposed to the typical aphasia patient, the pattern of language cortex reorganization appeared to mirror the same pattern. Notably, our patient was in the late subacute phase (4 months) at the end of iTBS intervention, yet the neural regeneration pattern in his language-related cortices seemed to approach characteristics of chronic phase aphasia patients, suggesting that prolonged iTBS intervention may accelerate the neural plasticity process in language-related cortices. In the chronic phase follow-up, the patient perceived his language performance as still below that of healthy individuals, although his language scale assessment results was nearly normal. fNIRS scanning of resting-state functional connectivity showed lower functional connectivity in his language-related cortices. A review of resting-state brain functional imaging studies in post-stroke aphasia summarized that functional connectivity partially normalizes in the subacute phase but remains low in chronic phase patients, indicating persistent covert language impairments [22]. This suggests that, despite the lack of abnormal clinical evidence on clinical language scales, neuroimaging can reveal the presence of covert aphasia.

This study has some limitations. It should be noted that this case report includes only one patient, hence the observed neuroimaging changes might not represent the general population. It should also be noted that our manuscript cannot conclusively verify that the observed recovery was due exclusively to the iTBS intervention. The improvement in the patient's condition could also be attributed to concurrent speech therapy or spontaneous recovery over time. Given that this study involved only one patient and lacked a control group, any causal relationship between iTBS and observed recovery is merely speculative on our part and cannot be definitively confirmed within the scope of this case report.

## 4 Conclusion

In conclusion, this case report illustrates a positive potential of using iTBS intervention for language improvement to facilitate aphasia recovery. Notably, this case involves a right hemisphere stroke, and the patient experienced a significant and sustained language improvement, supported by neuroimaging evidence of cortical reorganization and neural plasticity. The case underscores the importance of non-invasive brain stimulation in the cortical reorganization and neural plasticity process in stroke patients and offers valuable insights into the neural plasticity mechanisms of aphasia patients with right hemisphere damage.

## Ethics statement

The patient referred to in this paper agreed to its publication. The patient gave informed consent before participating in the study. The study was approved by the Ethics Committee of the Huashan Hospital Affiliated to Fudan University (No. 2019-512).

## Funding

This research was funded by the 10.13039/501100012166National Key R&D Program of China (No. 2018YFC2001604, 2018YFC2001700), 10.13039/501100001809National Natural Science Foundation of China (No. 82272607), Shanghai Disabled Persons' Federation Research Fund (No.2023ZC1021), Shanghai Healthcare System Key Support Discipline Construction Project (No.2023ZDFC0304).

## Data availability statement

The data presented in this study are available on request from the corresponding author.

## CRediT authorship contribution statement

**Haozheng Li:** Writing – original draft, Investigation, Formal analysis, Conceptualization. **Shunjuan Fan:** Writing – original draft, Data curation. **Yi Wu:** Writing – review & editing, Resources, Conceptualization. **Dongxiang Fang:** Visualization, Methodology. **Ruiping Hu:** Writing – review & editing, Writing – original draft, Software, Funding acquisition, Conceptualization. **Rongrong Lu:** Writing – review & editing, Resources, Methodology.

## Declaration of competing interest

We declare that we have no financial and personal relationships with other people or organizations that can inappropriately influence our work, there is no professional or other personal interest of any nature or kind in any product, service and/or company that could be construed as influencing the position presented in, or the review of the manuscript entitled.
